# Lateralized mechanisms for encoding of object. Behavioral evidence from an animal model: the domestic chick (*Gallus gallus*)

**DOI:** 10.3389/fpsyg.2014.00150

**Published:** 2014-02-24

**Authors:** Rosa Rugani, Orsola Rosa Salva, Lucia Regolin

**Affiliations:** ^1^Department of General Psychology, University of PadovaPadova, Italy; ^2^Center for Mind/Brain Sciences, University of TrentoTrento, Italy

**Keywords:** number cognition, lateralization, counting, number sense, arithmetic, addition, subtraction, domestic chick

## Abstract

In our previous research we reported a leftward-asymmetry in domestic chicks required to identify a target element, on the basis of its ordinal position, in a series of identical elements. Here we re-coded behavioral data collected in previous studies from chicks tested in a task involving a different kind of numerical ability, to study lateralization in dealing with an arithmetic task. Chicks were reared with a set of identical objects representing artificial social companions. On day 4, chicks underwent a free-choice test in which two sets, each composed of a different number of identical objects (5 vs.10 or 6 vs. 9, Experiment 1), were hidden behind two opaque screens placed in front of the chick, one on the left and one on the right side. Objects disappeared, one by one, behind either screen, so that, for example, one screen occluded 5 objects and the other 10 objects. The left-right position of the larger set was counterbalanced between trials. Results show that chicks, in the attempt to rejoin the set with the higher number of social companions, performed better when this was located to the right. However, when the number of elements in the two sets was identical (2 vs. 2, in Experiment 2) and they differed only in the coloration of the objects, this bias was not observed, suggesting a predisposition to map the numerical magnitude from left to right. Future studies should be devoted to the direct investigation of this phenomenon, possibly employing an identical number of mono-chromatic imprinting stimuli in both conditions involving a numerical discrimination and conditions not involving any numerosity difference.

## Introduction

Since Aristotle argued that “logos” is the essence of the human mind, logic and language were considered strictly connected (Houndé and Tzourio-Mazoyer, 2003; Vallortigara et al., 2010a,b; Vallortigara, 2012). From this perspective, all cognitive abilities, and especially mathematical thinking, were believed to be firmly related to language. This is likely to be correct for symbolic mathematical capacity (Carey, 2004). Indeed, the ability to represent number and selected numerical concepts, such as real numbers, logarithms, and square roots, is only performed by a subset of human beings, who have received specific mathematical education. Nonetheless, human adults are also able to master some numerical tasks when, under specific experimental conditions, language use is prevented (Cordes et al., 2001). This non-verbal “number sense” (all those calculations that could be solved in the absence of numerical words) can be found, for example, in tasks requiring individuals to add two sets of dots presented sequentially and to choose between a correct and an incorrect alternative. In this kind of task, both college students and rhesus monkeys (*Macaca mulatta*) are quicker and more accurate at selecting the greater of two numbers when the numerical distance between them is larger than when it is smaller (this is referred to as the Distance Effect). They also perform better in distinguishing between two small numbers compared to two larger numbers when the numerical distance is equal (this is referred to as the Magnitude Effect). Such a similarity in performance suggests that humans share a numerical processing mechanism with other animal species (Cantlon and Brannon, 2007).

Although this is the most direct evidence of an ancestral numerical mechanism shared by humans and non-humans, other supporting data have been obtained from non-human creatures (reviews in Vallortigara et al., 2010a,b). Rhesus monkeys (*Macaca mulatta*; Brannon and Terrace, 1998; Merritt et al., 2009), hamadryas baboons (*Papio hamadryas*), squirrel monkeys (*Saimiri sciureus;* Smith et al., 2003) and brown capuchin monkeys (*Cebus apella;* Judge et al., 2005) were able to master numerical tasks involving numbers up to 9, showing that discrimination of a numerical comparison depends on the ratio of the to be discriminated numbers (see also Call, 2000; Call, for evidence of numerical competence in orangutangs, *Pongo pygmaeus*). Some studies have shown that numerical cognition is not just a prerogative of primates, but that it can be found also in a non-mammalian species, for example in the Class Aves. Simple quantity discrimination (preference for the bigger between two sets of food items) has been demonstrated in robins (*Petroica longipes*) (Garland et al., 2012). An African gray parrot (*Psittacus erithacus*) even learned to use labels to order numbers up to 8 (Pepperberg, 2012).

Evidence of number discrimination ability has been obtained also in very young birds (Rugani et al., 2008). Newborn chicks (*Gallus gallus*) were reared with two stimuli, each characterized by a different number of elements. Food was found in proximity of one of the two stimuli. Subjects were then tested with stimuli depicting novel elements representing either the numerosity associated or not associated with food. Chicks approached the number associated with food in the 2 vs. 3, 2 vs. 8, 6 vs. 9, 8 vs. 14, 4 vs. 6, and 4 vs. 8, 5 vs. 10, and 10 vs. 20 comparisons, and did so even when quantitative cues were unavailable or controlled (Rugani et al., 2013a). Spontaneous number discrimination was demonstrated also by taking advantage of chicks' sensitivity toward the fine visual characteristics of their own imprinting object. Chicks reared with groups of artificial stimuli of different numerousness prefer to approach, during a subsequent test, the set containing the higher number of imprinting objects in the comparisons 1 vs. 2, 1 vs. 3, and 2 vs. 3 (Rugani et al., 2010a). Moreover, when chicks are presented with sets of 2 vs. 3, 1 vs. 4, and 2 vs. 4 imprinting objects disappearing one-by-one, each set behind one of two screens, they spontaneously inspected the screen occluding the larger set, even when the continuous variables (total surface area or contour length) were controlled for (Rugani et al., 2009, 2013b,c). Nevertheless, when chicks were presented with comparisons between large numbers of objects (5 vs. 10 or 6 vs. 9), they succeeded only if non-numerical and numerical cues were both available (Rugani et al., 2011a).

From these and other evidence (see Vallortigara et al., 2010a,b for a review) it seems that numerical competence did not emerge *de novo* in linguistic humans, but has been likely built on precursor systems also available in non-human animals (Dehaene, 1997; Carey, 2009).

In the field of numerical cognition, another prerogative that, up to now, was considered to be uniquely human is the tendency to orient numbers from left (small numerical values) to right (large numerical values; Galton, 1880; Dehaene, 1993; Fias and Fischer, 2005; Bueti and Walsh, 2009). An example of this is provided by the SNARC (Spatial Numerical Association of Response Codes) effect, in which humans respond faster to smaller numbers with the left hand and to larger numbers with the right hand (Dehaene et al., 1993). Also, when adult humans attempted to generate numbers at random they were influenced by lateral head turns: when the participants were facing left they produced relatively small numbers, whereas when facing right they tended to produce larger numbers (Loetscher et al., 2008). Patients with left-sided visuospatial neglect, typically due to damage to the right parietal lobe, bisected the numerical interval with a systematic bias toward larger numbers (Zorzi et al., 2002). In addition to that, evidence supports a universal left-sided attention bias in number space: healthy subjects required to estimate the midpoint of a numerical interval show a systematic error, consistently misplacing the midpoint slightly to the left of its actual position (Göbel et al., 2001).

Many studies suggested that these lateralization effects emerge as a result of exposure to formal instruction (Shaki et al., 2009), since scholar education could reduce or even reverse the SNARC effect in cultures that read from right to left (Zebian, 2005; Shaki and Fischer, 2008; Shaki et al., 2009). However, the origins of this asymmetry, and particularly the degree to which it depends upon cultural experience, remains elusive. Recently de Hevia et al. (2008), de Hevia and Spelke (2010) have demonstrated that a predisposition to relate number to space develops early in life, before the acquisition of language. They have showed that 8-month-old infants transfer the discrimination of an ordered series of numerosities to the discrimination of an ordered series of line lengths. Infants therefore have an intrinsic preference for numbers and lengths that are positively related. Even more suggestive are the data that illustrate a tendency to represent numerical magnitudes as oriented from left to right in non-human animals (Rugani et al., 2007, 2010b, 2011b). Two bird species, domestic chickens and Clark's nutcrackers (*Nucifraga Columbiana*) were trained to select a target element in a series of identical ones, sagittaly oriented with respect to the bird's starting point. Birds were then tested with a series, identical to the first one, but rotated by 90°, so that the target could be identified either from the left or from the right end of the series. Both species selected the target with respect to the left end, suggesting that a disposition to map the numerical magnitude from left to right may originate from a prelinguistic precursor. Nevertheless, the leftward preference could be related to a general bias in the allocation of attention. In humans this phenomenon has been named “pseudoneglect” and reflects the fact that we primarily attend to the objects in the left side of space (Bowers and Heilman, 1980; Jewell and McCourt, 2000). Again, this is not a prerogative of human beings, in fact a selective allocation of attention to the left hemifield can be found also in birds during free foraging (Diekamp et al., 2005; Chiandetti, 2011) and in a comparative version of the line bisection task (Regolin, 2006). Somewhat similar phenomena favoring the left hemifield have been described also for amphibians (Vallortigara et al., 1998; Vallortigara and Rogers, 2005), suggesting a common mechanism shared by phylogenetically distant species.

Differently, an advantage for processing bigger numerosity, presented in the right hemispace, could not be explained as by product of selective left-sided attentional bias. In one of our studies, newly-hatched domestic chicks were reared for 3 days with a group of identical artificial imprinting objects. At test when animals were presented with sets of 5 vs. 10 (or 6 vs. 9) objects disappearing behind one of two identical screens, they spontaneously inspected the screen occluding the larger set (Rugani et al., 2011a). Across subsequent trials the larger set was made to disappear either behind the screen located to the left or to the right (with respect to the bird's starting position), offering the possibility to test for the presence of lateralization effects. Here we reanalyze the behavior of the subjects, to investigate if the performance is affected by the left-right position of the two sets. If a tendency to represent numerousness from left to right does exist in this species, we would expect an advantage when searching for the larger number of social companions if this is located to the right side.

## Experiment 1

In previous studies we reported, in two bird species, a preference to map numbers from left to right, suggesting a lateralized representation of number space (Rugani et al., 2007, 2010a, 2011a,b).

Here we investigate this phenomenon by observing chicks' choice between a larger vs. a smaller group of artificial social companions (i.e., objects chicks have been familiarized to through exposure). Chicks are motivated to reach the larger group of objects. If smaller vs. larger numerosities are spatially mapped from left to right then we should expect chicks to be better at responding to the larger group when located on the right. Notably, such a finding would not be explained by the hypothesis of attentional facilitation for the left hemispace.

### Materials and methods

#### Subjects and rearing conditions

For the present experiment we re-coded behavioral data from a sample of 36 female domestic chicks (*Gallus gallus*). Being the attractor a social stimulus we employed solely female chicks, since female chicks are more motivated than males to retrieve a social companion (Regolin et al., 2005). Data were originally collected by Rugani et al. (2011a). Subjects were obtained from a local commercial hatchery (Agricola Berica, Montegalda, Vicenza, Italy) when they were only a few hours old. On arrival at the laboratory, each chick was singly housed in standard metal home cage (28 cm wide × 32 cm long × 40 cm high) at controlled temperature (28–31°C) and humidity (68%), with food and water available *ad libitum* in transparent glass jars (5 cm in diameter, 5 cm high) placed at corners of the home cage. The cages were constantly (24 h/day) lit by fluorescent lamps (36 W), located 45 cm above the floor of the cages. Each chick was reared together with an imprinting stimulus composed of five identical objects. These were the same for all chicks and consisted of two-dimensional, about 1 mm thick, red plastic squares (2.5 × 2.5 cm). Each object was suspended in the center of the cage by a fine thread, at about 4–5 cm from the floor, so that they were all located at about chicks' head height.

Previous studies have shown that this kind of object is very effective in producing social attachment through filial imprinting in chicks (Rugani et al., 2009, 2010a, 2011a, 2013b).

Chicks were reared in these conditions from the morning (11 am.) of the 1st day to the morning (12 am.) of the 3rd day of life, when each subject singly underwent training and, about 2 h later, testing. In the time between training and testing, chicks were placed back to their own cage with their imprinting objects.

At test, different numerical comparisons were used for different groups of chicks. Eighteen chicks underwent the 5 vs. 10 comparison. These chicks were divided in two experimental groups, depending on the stimuli employed during testing. For the “no-control group” (*N* = 10), the original dimensions of the imprinting squares (2.5 × 2.5 cm) were maintained, so that both sets were composed of identical squares. In the “controlled-stimuli group” (*N* = 8), the set of 10 elements again comprised squares which dimensions were identical to those used during imprinting. On the contrary, the set of five elements comprised larger sized squares, balanced for either the overall area or for the overall perimeter. In fact, for half of the chicks of the “controlled-stimuli group” the dimensions of each square in the set of five elements were computed in order to match the overall perimeter of the set of 10 elements (with squares measuring 5.00 × 5.00 cm each). For the other 4 chicks, the set of five elements had the same overall area of the set of 10 elements (with squares measuring 3.54 × 3.54 cm each).

Other 18 chicks were tested with the comparison 6 vs. 9. As for the first numerical comparison, 10 chicks were tested with stimuli in which continuous variables co-varied along with numerousness. For this “no-control group,” 15 identical squares measuring 2.5 × 2.5 cm were used. Again, the remaining eight chicks were tested with stimuli in which continuous variables were equated between the two sets. For half of the chicks of the “controlled-stimuli group” the dimensions of the squares in the set of six objects were computed to equate the overall perimeter of the set of nine objects (with squares measuring 3.75 × 3.75 cm each). The other 4 chicks were presented with sets equated in the overall area (with squares measuring 3.06 × 3.06 cm each).

#### Apparatus

**Figure 1:**
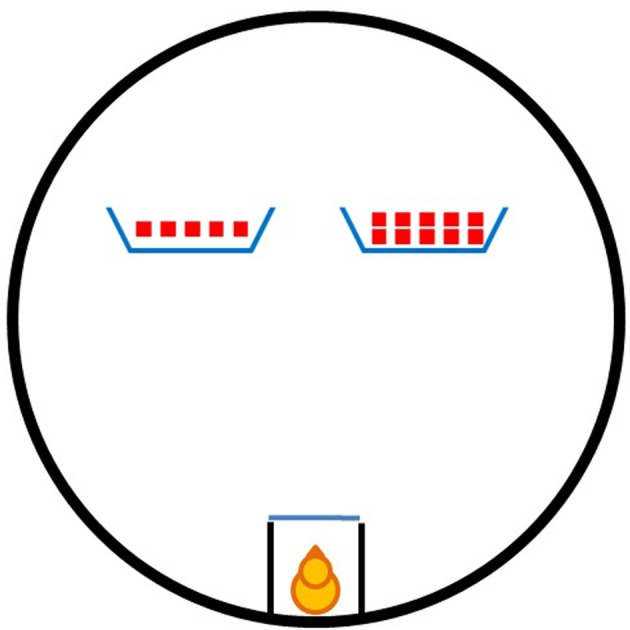
**The apparatus employed in both experiments.** The holding box and the two screens are here represented in the arena just as they were during the testing session.

Training and testing took place in an experimental room located near the rearing room. In the experimental room temperature and humidity were controlled (respectively, at 25°C and 70%). The room was kept dark, except for the light coming from a 40 W lamp, placed about 80 cm above the floor of the apparatus. The experimental apparatus (Figure 1) consisted of a circular arena (95 cm in diameter and 30 cm outer wall height) with the floor uniformly covered by a white plastic sheet. Within the arena, adjacent to the outer wall, was a holding box (10 × 20 × 20 cm), in which each subject was confined shortly before the beginning of each trial. The box was made of opaque plastic sheets, with an open top allowing the insertion of the chick before each trial. The side of the holding box facing the center of the arena consisted of a removable transparent glass partition (20 × 10 cm), this allows the subjects, while confined, to see the inner of the arena. During the training phase a single opaque cardboard screen (16 × 8 cm; with 3 cm sides bent back to prevent the chicks from seeing objects hidden behind the screen) was used, positioned in the center of the arena, in front of and 35 cm away from the front of the holding box. During testing, two opaque cardboard screens (16 × 8 cm), identical in color and pattern (i.e., blue colored with an orange “X” on them), were positioned in the center of the arena, symmetrically with respect to the front of the confining box (i.e., 35 cm away from it, and 20 cm spaced apart from one another).

#### Procedure

***Training.*** On day three of life, at around 12.30, chicks underwent a preliminary training session. Each chick, together with a single object, identical in color and dimension to the squares composing its imprinting stimulus, was placed within the testing arena, sitting in front of the starting box and facing the screen. The object was held from above by the experimenter (not visible to the chick), via a fine thread, and kept suspended 3–4 cm over the floor, at an intermediate position between the holding box and the screen (about 15 cm away from the screen). This initial phase lasted for 5 min, over this period the chick was free to move around and get acquainted with the environment. Thereafter, the experimenter slowly moved the object toward the screen, and then behind it, until it disappeared from the chick's sight. This procedure was repeated a few times, until the chick started to follow the object behind the screen as soon as it was made to disappear. Thereafter, the chick was confined within the holding box, from where it could see the object being moved behind the screen. As soon as the object had completely disappeared from sight, the chick was set free in the apparatus by lifting the transparent frontal partition. Every time the chick rejoined the object, as a reward, it was allowed to spend a few seconds with it. The whole procedure was restarted and the training ended when the chick had rejoined the object three consecutive times. On average, about 15 min were required to complete the training for each chick.

***Testing.*** Testing took part 2 h after the end of training and it was composed of 20 trials. At the beginning of each trial, the chick was confined to the holding box with the transparent partition in place, from where it could see the two screens in the arena. The chick was presented with only one element at a time and could not see either set as a whole. Every element of the first set was placed about 10 cm from the front of the holding box and then it was made to disappear behind one of the screens. Immediately after it disappeared the next element was introduced into the arena. In this way, all the elements of the first set were made to disappear one by one behind the same screen. Then, the identical procedure was repeated for the second set behind the other screen. Each element was kept in front of the starting box for 3 s and then it took 3 s to be moved back behind the screen (6 s overall). About 2 s elapsed from the disappearance of one object and the appearance of the next one. 3 s after the disappearance of both sets, the transparent partition was removed and the chick was left free to move within the arena. In this way the whole procedure of stimuli presentation lasted about 121 s for each trial. The order the two sets were presented (which one was presented first) as well as the position where they disappeared (left or right screen) was counterbalanced within each chick's testing trials. At the end of stimuli presentation the chick was released in the arena by removing the frontal transparent partition and was allowed to look behind either of the two screens. A choice for one of the screens was defined as when the chick's head had entered the area behind the screen. Only the choice for the first screen visited was scored and thereafter the trial was considered over. At the end of each trial, as reward, the chicks were allowed to spend a few seconds with their “social companions” behind the screen chosen. The behavior of the chicks was entirely video-recorded and it was scored blind both online and later offline.

If the chick did not approach either screen within 3 min, the trial was considered null and void and it was repeated immediately afterwards. Whenever the chick failed to respond also at the second attempt of performing the trial, that trial was considered as null and recorded as such, this means that chicks could score less than 20 valid trials. In the first experiment two chicks scored 19 valid trials and two other chicks scored 18 valid trials, the remaining 14 subjects scored all 20 valid trials.

### Data analysis and results

Previous literature showed that in this sort of task chicks have a clear tendency to approach the screen hiding the larger group of social companions (Rugani et al., 2009, 2011a,b, 2013a; Fontanari et al., 2011). Thus, we will henceforth define as “correct” the choice for the screen hiding the higher number of imprinting objects. We will similarly define the more numerous group of social companions as “target group.”

This tendency to approach the larger group is also true for what concerns the performance of the group of chicks re-coded here (Rugani et al., 2011a). When the performance “no-control group” was compared with the chance level, it resulted that subjects preferentially chose the screen hiding 10 objects over the screen hiding 5 objects [*n* = 10; Mean = 69.423, s.e.m. = 2.693; one-sample *t*-test: *t*(_9_) = 7.213; *p* < 0.001], or in the comparison 6 vs. 9, the screen hiding 9 objects over the screen hiding 6 objects [*N* = 10; Mean = 66.777, s.e.m. = 2.693; *t*(_9_) = 7.619; *p* < 0.001]. Nevertheless the capability to solve proto-arithmetic calculations seems to be possible solely when numerical and quantitative cues were contemporary available. When the perimeter or the area were controlled for (“control group”) we did not find any significant preference [5 vs. 10: *N* = 8; Mean = 53.263, s.e.m. = 2.320; *t*(_7_) = 1.407; *p* = 0.202; 6 vs. 9: *n* = 8; Mean = 50.361, s.e.m. = 3.747; *t*(_7_) = 0.096; *p* = 0.962].

A laterality index was calculated to represent the percentage of right-sided correct choices on the overall number of correct choices, according to the formula:

(Number of correct choices when the target group was on the right screen/Total number of correct choices) × 100.

The laterality index can assume values ranging from 0 (all correct choices performed when the target group is behind the left screen) to 100 (all correct choices performed with the target group behind the right screen); a value of 50 indicates an equal number of correct choices on both sides (chance level).

The laterality index was analyzed by a 2 × 2 ANOVA with (between-subjects factors) Numerical Comparison (“5 vs. 10” and “6 vs. 9”) and Control for Continuous Variables (“control” and “no control”). As since no significant effect for the factor Numerical Comparison [*F*_(1, 32)_ = 1.910; *p* = 0.177] nor an interaction between this factor and the Control for Continuous Variables [*F*_(1, 32)_ = 0.017; *p* = 0.897] was detected, data were collapsed in all further analyses and comparisons between groups were performed by an independent sample *t*-test for unequal variances (Ruxton, 2006). Laterality effects were assessed comparing the laterality index to chance level via one-sample *t*-tests.

**Figure 2:**
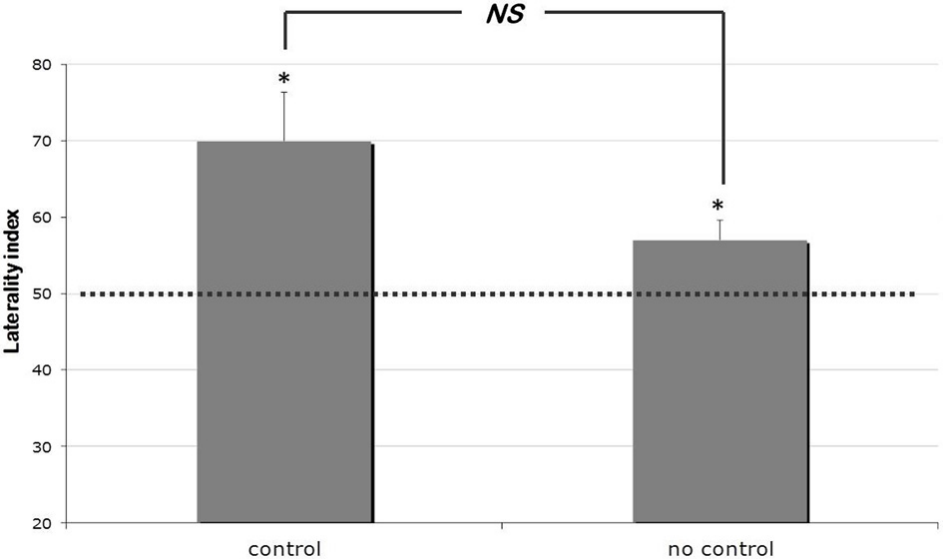
**Results of Experiment 1.** Percentages of laterality index (means ± s.e.m.) scored by control and no-control group of chicks. The dotted line (*y* = 50) represents chance level. ^*^ indicates *p* < 0.05.

Overall, chicks were significantly lateralized and performed a higher percentage of correct choices when the target was on the right position [*t*_(35)_ = 3.777, *p* = 0.001, mean = 63%, s.e.m. = 3%]. Such bias appeared to be more pronounced for chicks of the “control” rather than of the “no control” group (see Figure 2). However, only a marginally non-significant difference was detected between these two groups [*t*_19.99_ = 1.949, *p* = 0.065; mean of the “no control” group = 70%, s.e.m. = 6%; mean of the “control” group = 57%, s.e.m. = 3%, Cohen's *d* = 0.642]. Marginally non-significant results should of course be treated with caution given their difficult interpretation. Nevertheless, in the light of the pronounced difference between the mean score observed in the two groups, we run a separate analysis comparing the “no control” group with chance level. This allowed us to verify that a significant lateralization effect could be detected even in the group for which continuous variables were not controlled [*t*_19_ = 2.53, *p* = 0.02].

## Experiment 2

In Experiment 1 we provide a first evidence in a non-human species of an advantage when the larger set is found to the right side of the subject. This bias could be due to an effect specific of numerical processing, or rather to a non-numerical preference when searching for social attractors on the right side. To control for this alternative explanation, in the present experiment, we analyzed the behavior of chicks tested according to the same paradigm, but with equal numbers of objects disappearing behind each screen. If the bias highlighted in Experiment 1 has a non-numerical basis, we would expect it to appear also here, when choice is not based on numerical cues as identical numbers of items are presented to the left and to the right side.

Both sets used in Experiment 2 were composed of two objects (i.e., the comparison was of 2 vs. 2). The numerosity of each set, hence the overall number of objects present, was smaller than in Experiment 1. Rearing conditions, however, were very similar in that chicks in both experiments were exposed to multiple (i.e., five or six) objects.

### Materials and methods

#### Subjects and rearing conditions

For the present experiment we analyzed the behavior of a sample of 12 female domestic chicks (*Gallus gallus*). Behavioral data were originally collected by Fontanari et al. (2011). The experiment that we have re-coded here was originally designed to investigate if chicks were able to use property information (e.g., color) for object individuation, exploiting chicks' spontaneous tendency to approach the larger group of familiar objects. For this reason imprinting stimuli differed from Experiment 1, being composed of three green squares and three yellow squares (4 × 4 cm). Beside that, rearing conditions were identical to those previously described. This should not cause any difficulty for the comparison of the results of the present experiment and of Experiment 1, where objects of identical color were used. Indeed, for the chicks of Experiment 2 objects of both colors were familiar, in a comparable way with respect to Experiment 1, because both have been used during rearing and were treated as imprinting objects (Rugani et al., 2010a).

#### Training stimuli and procedure

Testing stimuli were green and yellow squares (4 × 4 cm). At each training trial only a single square (either a yellow one or a green one) was used. During training the two stimuli were used the same number of times. All the other training conditions were exactly the same described for the Experiment 1.

#### Testing stimuli and procedure

Test stimuli were identical to those employed during training. At each testing trial two pairs were sequentially presented (a low-variety and a high-variety pair). For the low-variety pair two identical squares (yellow + yellow or green + green) were used. For the high-variety pair two squares of a different color (yellow + green) were employed. The presentation of each pair proceeded as follows: the two objects were made to simultaneously appear from one screen, coming in front of the chick confined in the holding box and then made to slowly disappear behind the same screen. The whole procedure took approximately 20 s. After a delay of 5 s, the chick was set free within the arena. Ten test trials were administered to each chick.

The use in the two pairs of the color (yellow or green) of the objects was randomized between subjects, whereas the order of presentation of the two pairs as well as which screen concealed which pair were counterbalanced within subjects across subsequent trials.

No subjects performed null trial in this Experiment.

### Data analysis and results

In this experiment chicks were not presented with a numerical discrimination, but rather with the choice between approaching either a screen hiding two identical social companions (low-variety pair), or a screen hiding two social companions differing in color from one-another (high-variety pair). We arbitrarily defined the high-variety pair as the target group. In order to compute a laterality index we thus applied the formula:

(Number of choices when the target group was on the right screen/Total number of choices for the target object) × 100.

A one-sample *t*-test was used to compare the laterality index with chance-level (i.e., with the value of 50%, indicating absence of lateralization). Contrary to what observed in Experiment 1, in the present experiment we were unable to detect any significant departure from chance level [*t*_(11)_ = 0.379, *p* = 0.712, mean = 52%, s.e.m. = 6%].

It should be noted that the absence of a significant effect in this case could be related to the minor number of subjects tested in Experiment 2. To assess this objection we have run two different analyses. First of all we computed the minimum number of subjects that would be required to reach a statistically significant effect, given the effect size observed in Experiment 1. A power analysis (G^*^Power 3.1 software) revealed that, assuming the standard power value of 0.8, at least 14 subjects would be required in a one-tailed *t*-test. That is, two subjects more than those employed in Experiment 2. The sample size of Experiment 2 is not far way from the desired N, nevertheless on the basis of this result we have to recognize that is not possible to rule out lack of statistical power as an explanation. Also, the results of the power analysis are crucially dependent on the arbitrary value assigned to the “power” parameter, and more conservative values would increase the dimension of the required sample size. However, these computations are of course based on the assumption that the same effects size computed for Experiment 1 applies also to Experiment 2. Another interesting approach is to compute the minimum sample size needed to reach significant departure from chance level, based on the values of Mean and SD actually observed in Experiment 2. This revealed that the number of subjects that would be required to reach a statistically significant effect in this Experiment would be of 620, greatly exceeding the sample size of Experiment 1. This speaks against the possibility to obtain drastically different results by increasing sample size of two units.

To conclude even if this second Experiment is not characterized by a strong power, nonetheless it seems to suggest that a number-space association could be there in this kind of task. On the grounds that non-significant results must be interpreted with caution, it is not possible to unequivocally conclude that our results reflect a precursor of the left-to-right mental number line orientation, but this investigation will be one of the most relevant scientific challenges in this field of research.

## General discussion

Experiment 1 allowed us to detect a rightward bias, evident when domestic chicks are required to search for the larger number of objects in the comparisons 5 vs. 10 and 6 vs. 9. In contrast, Experiment 2 revealed that, when no numerical discrimination is involved in the task, chicks tested in the same apparatus and with a similar procedure to that described for Experiment 1, do not reveal any directional bias. This difference could be due to a number of reasons. First of all, set numerosities involved in Experiment 1, 2 were rather different, with large numerosities being employed in the first and small numerosities being employed in the second experiment. However we have no reasons to believe that small or large numbers of social companions trigger for qualitatively different processing. Moreover similar rearing conditions were used in the two experiments, exposing chicks in both cases to multiple objects. This procedure would activate the same cognitive system for the processing of both small and large numbers (Rugani et al., 2013a,b,c). A second issue concerns whether a preferential choice is or is not expressed by subjects for one of the two sets. Fontanari et al. (2011) (where from data of Experiment 2 come) reported lack of preference between two identical vs. two different objects. Absence of any significant lateralization in Experiment 2 may therefore depend on the lack of preference for one of the two sets. This hypothesis though would not be consistent with evidence provided in Experiment 1 of the present paper. In fact, chicks in the study of Rugani et al. (2011a) (where from data of Experiment 1 come) did not discriminate sets of 6 vs. 9 and 5 vs. 10 objects when continuous variables were controlled for. Nevertheless, in Experiment 1 a clear lateralization emerged for chicks tested in such condition. Indeed, chicks of the “control” condition tended to display an even more pronounced rightward bias than chicks in the “no control” condition. Both in Experiment 2 and in the “control” condition of Experiment 1 chicks did not show a significant preference for one of the two sets. Nevertheless, only when the two sets differed in numerosity, such as in Experiment 1, chicks emitted a higher number of correct choices if the larger set was on their right side.

It seems that a bias can be observed only when chicks have to choose between sets differing in numerosity. This evidence would support previous findings that animals map numerical values onto space, though it would demand an explanation beyond the hypothesis of attentional facilitation for the left hemispace. Further research is warranted for understanding this phenomenon, the effect should be replicated with other numerosities and with new control conditions, but most interestingly, new experiments should probe, within a same paradigm, both an advantage to respond to large numbers located to the right side as well as to small numbers located to the left side.

Here we have shown that chicks, in the attempt to rejoin the set with the higher number of social companions, performed better when this was located to their right side. This bias is reminiscent of the well-known phenomenon of the left-to-right orientation of number line in our species. Originally this orientation was thought to be dependent on cultural factors, such as the reading direction, making it implausible to observe a similar phenomenon in non-human animals (Dehaene et al., 1993). This interpretation is also supported by the fact that the association of smaller numbers with left space and larger numbers with right space is stronger in bilingual subjects after reading a Russian text (that is read from left to right) than after reading an Hebrew text (that is read from right to left; Shaki and Fischer, 2008). More recent investigations, however, suggest that reading habits themselves are unlikely to be the only origin of this spatial-numerical arrangement (Fischer and Brugger, 2011). For example, it is possible to reverse the spatial association for numbers merely by instructing observers to think of numbers as either indicating lengths on a ruler or time on a clock face (which have opposite horizontal mappings for small and large digits; Bächtold et al., 1998; Vuilleumier et al., 2004). Moreover, developmental studies suggests that preschool children already explore objects more efficiently when they are numbered in ascending order from left to right (Opfer and Furlong, 2011). Using a manual bisection paradigm, with lines flanked by arrays of dots, 5-year-old children showed the same bias of 7-year-old children and adults, indicating that the left-to-right mapping of numbers into space could emerge spontaneously and independently of formal instruction (de Hevia and Spelke, 2009).

Evidence suggestive of a left-to-right numerical orientation has been recently obtained also in non-human species. Domestic chicks and Clark's nutcrackers, trained to select a target element in a sagitally-oriented series and tested with a rotated series, identified as correct solely the element from the left end of the series (Rugani et al., 2007, 2010b, 2011b). This phenomenon, however, could be linked to a general bias for allocating attention in the left emispace, rather than to a specific lateralization of numerical representation (Rugani et al., 2011b). Here, employing a completely different paradigm, we reported a rightward bias that emerges when domestic chicks are required to search for the larger number of objects, in the comparisons 5 vs. 10 and 6 vs. 9 (Experiment 1). Such a bias was not found when the numerousness of the two sets were equated, in the comparison 2 vs. 2 (Experiment 2). Obviously, such an advantage for the right-hemispace cannot be explained as a byproduct of a leftward attentional prioritization. Although further evidence is necessary, we believe that the results presented in this paper provide the first evidence suggesting an orientation effect of purely numerical origin.

Interestingly enough, it is well known that in this species the level of lateralization is determined by the exposure of embryos to light during a critical period (from day 17 to 21 of incubation). Chicks hatched from light incubated eggs are strongly lateralized, whereas the lateralization is largely prevented in dark-incubated chicks (Daisley et al., 2009; Chiandetti, 2011). All chicks used in these Experiments came from a commercial hatchery, where eggs were maintained in darkness. However, sometimes the light was turned on in order to guarantee the routine maintenances, reducing the control over the degree of lateralization caused to the embryos. We thus consider these subjects as poorly lateralized, but, due to the not perfectly controlled incubation conditions, we are currently unable to draw strong conclusions about the role of light-exposure in this lateralization effect. This issue could be better investigated in future experiments with chicks obtained from light vs. dark laboratory-incubated eggs.

Overall these data suggest that a disposition to map the numerical magnitude from left to right may originate from a prelinguistic precursor. The phenomena associated with basic numerical competence seem to be rooted in biological primitives that can be explored also in very young animals. Some sort of a Kantian “a priori” intuition that precedes and structures how animals (human and non-human) experience the environment.

### Conflict of interest statement

The authors declare that the research was conducted in the absence of any commercial or financial relationships that could be construed as a potential conflict of interest.
